# Mitochondrial Bioenergetics and Fiber Type Assessments in Microbiopsy vs. Bergstrom Percutaneous Sampling of Human Skeletal Muscle

**DOI:** 10.3389/fphys.2015.00360

**Published:** 2015-12-18

**Authors:** Meghan C. Hughes, Sofhia V. Ramos, Patrick C. Turnbull, Ali Nejatbakhsh, Brittany L. Baechler, Houman Tahmasebi, Robert Laham, Brendon J. Gurd, Joe Quadrilatero, Daniel A. Kane, Christopher G. R. Perry

**Affiliations:** ^1^Muscle Health Research Centre, School of Kinesiology and Health Science, York UniversityToronto, ON, Canada; ^2^Department of Kinesiology, University of WaterlooWaterloo, ON, Canada; ^3^School of Kinesiology and Health Studies, Queen's UniversityKingston, ON, Canada; ^4^Department of Human Kinetics, St. Francis Xavier UniversityAntigonish, NS, Canada

**Keywords:** mitochondria, respiration, permeabilized fiber, muscle biopsy, blebbistatin

## Abstract

Microbiopsies of human skeletal muscle are increasingly adopted by physiologists for a variety of experimental assays given the reduced invasiveness of this procedure compared to the classic Bergstrom percutaneous biopsy technique. However, a recent report demonstrated lower mitochondrial respiration in saponin-permeabilized muscle fiber bundles (PmFB) prepared from microbiopsies vs. Bergstrom biopsies. We hypothesized that ADP-induced contraction (rigor) of smaller length microbiopsy PmFB causes a greater reduction in maximal respiration vs. Bergstrom, such that respiration could be increased by a myosin II ATPase-inhibitor (Blebbistatin; BLEB). Eleven males and females each received a 2 mm diameter percutaneous microbiopsy and a 5 mm diameter Bergstrom percutaneous biopsy in opposite legs. Glutamate/malate (5/0.5 mM)—supported respiration in microbiopsy PmFB was lower than Bergstrom at submaximal concentrations of ADP. 5 μM BLEB reduced this impairment such that there were no differences relative to Bergstrom ± BLEB. Surprisingly, pyruvate (5 mM)-supported respiration was not different between either biopsy technique ±BLEB, whereas BLEB increased succinate-supported respiration in Bergstrom only. H_2_O_2_ emission was lower in microbiopsy PmFB compared to Bergstrom PmFB in the presence of BLEB. Microbiopsies contained fewer type I fibers (37 vs. 47%) and more type IIX fibers (20 vs. 8%) compared to Bergstrom possibly due to sampling site depth and/or longitudinal location. These findings suggest that smaller diameter percutaneous biopsies yield lower glutamate-supported mitochondrial respiratory kinetics which is increased by preventing ADP-induced rigor with myosin inhibition. Microbiopsies of human skeletal muscle can be utilized for assessing mitochondrial respiratory kinetics in PmFB when assay conditions are supplemented with BLEB, but fiber type differences with this method should be considered.

## Introduction

The Bergstrom muscle biopsy technique has been an essential tool for the direct assessment of human skeletal muscle responses to a variety of physiological perturbations (Bergstrom, 1975). More recently, percutaneous microbiopsy needles have been adopted as an alternative approach to sample skeletal muscle in humans. Anecdotally, microbiopsies are often perceived as less invasive than Bergstrom given they obtain smaller samples and do not require an incision on the skin and fascia for the common procedure of sampling human vastus lateralis skeletal muscle. While these arguments have not been fully validated, interest in microbiopsies has increased nonetheless, particularly for the assessment of skeletal muscle metabolism (Hayot et al., 2005; Jorge et al., 2011; Krause et al., 2012; Votion et al., 2012). As such, validation of this methodology for metabolic assays in skeletal muscle is required given the potential influence of the sampling method on experimental results.

Permeabilized muscle fiber bundles (PmFB) are commonly used to assess mitochondrial bioenergetics in skeletal muscle. This technique requires careful separation of muscle fibers with fine forceps followed by chemical permeabilization with cholesterol-binding detergents (Kuznetsov et al., 2008; Pesta and Gnaiger, 2012; Perry et al., 2013). Surprisingly, it was recently reported that microbiopsies of pig skeletal muscle yielded lower mitochondrial respiration in PmFB which appeared to be positively correlated to needle diameter (Isner-Horobeti et al., 2014). While the exact cause of this lower respiration was not identified, it is possible that the smaller fiber length of PmFB from microbiopsies pose a challenge to preparing intact PmFB that can withstand the magnetic stirring during respirometric assessments. Loss of PmFB integrity could impair respiratory assessments given it has previously been established that preservation of mitochondrial structure and morphology are critical for optimizing respiratory assessments in PmFB (Veksler et al., 1987; Kuznetsov et al., 2008; Picard et al., 2011).

A key factor influencing PmFB intactness is a phenomenon of ADP-induced rigor, such that the very nature of assessing ADP-stimulated respiratory kinetics also induces contraction and disintegration *in vitro* (Ventura-Clapier and Vassort, 1985; Perry et al., 2011, 2013). Such PmFB contraction influences respiratory kinetics but can be prevented by the addition of Blebbistatin (BLEB), a myosin II ATPase inhibitor, to the assay media (Perry et al., 2011, 2012, 2013). Blebbistatin binds to the active site of subfragment 1 ATPase when ADP and phosphate are bound which stabilizes the intermediate state (Kovacs et al., 2004) lowers force production (Fedorov et al., 2007; Farman et al., 2008; Minozzo et al., 2012) and prevents shortening of muscle cell length (Fedorov et al., 2007; Farman et al., 2008; Ebrahim et al., 2013) during contraction. In fact, unpublished observations within our laboratory revealed drastic differences in mitochondrial respiration rates of PmFB in the presence (+BLEB) and absence (−BLEB) of BLEB from samples obtained using the microbiopsy technique. Moreover, microbiopsy PmFB appeared to be more prone to fiber disintegration and impaired respiratory kinetics following ADP-induced contraction than Bergstrom PmFB. If true, these observations would not only further highlight the importance of PmFB conformation when measuring mitochondrial function, but also suggest BLEB may be an effective tool for rescuing impaired respiration in microbiopsies.

The purpose of this investigation was to compare mitochondrial bioenergetics in samples obtained with the Bergstrom and microbiopsy techniques using BLEB as a tool to control the contractile state of PmFB *in vitro*, and furthermore, to characterize the fiber type composition of the samples obtained using each biopsy technique. We hypothesized that BLEB would increase respiration in microbiopsy PmFB *in vitro* by normalizing respiratory kinetics to Bergstrom PmFB. Likewise, we hypothesized that BLEB would result in similar mitochondrial H_2_O_2_ emission rates between microbiopsy and Bergstrom PmFBs. Finally, we expected fiber type composition analyses would be similar despite differences in biopsy size between both techniques.

## Materials and methods

### Human participants and muscle biopsies

Eleven healthy, recreationally active males (*n* = 5) and females (*n* = 6) were recruited to participate in this investigation. Their mean ± standard error of the mean (SEM) age, height, weight and BMI were 25.3 ± 0.6 years, 171.7 ± 2.4 cm, 70.6 ± 4.8 kg, and 23.8 ± 1.3 kg·m^−2^, respectively. All participants were non-smokers, free of disease and not taking prescription medications or supplements. Participants were given both oral and written information about experimental procedures before giving informed consent. All experimental procedures with human participants were approved by the Research Ethics Board at York University and conformed to the Declaration of Helsinki.

With the participant lying supine on a bed, a skeletal muscle sample was obtained from the lateral aspect of the right vastus lateralis by percutaneous needle biopsy technique using a spring-loaded 14 gauge (~1.5 mm) Medax Biofeather microbiopsy disposable needle (San Possidonio, MO, Italy) under local subcutaneous anesthesia (~2 ml of 2% xylocaine without norepinephrine). A 12 gauge (~2 mm) cannula was used to puncture the skin at ~30° from the surface to a depth of 2 cm and guide the needle to an additional depth of 2 cm longitudinally along the vastus lateralis. Four to five cuts (10–20 mg each) were sampled with the needle rotating ~30–40° between cuts over a period of ~1 min. Each piece was removed from the needle with sterile forceps or surgical blades before the subsequent cut was made. The first three samples were used for preparation of fiber bundles, and the remaining were used for fiber type analysis (described below). After applying a bandage to the ~2 mm diameter biopsy site, a second skeletal muscle sample was obtained from the left vastus lateralis using the Bergstrom needle biopsy technique (Bergstrom, 1975) with manual suction (Shanely et al., 2014). Three to four cuts (100–150 mg total) were sampled over a period of ~10 s. Approximately 40 mg of sample was used for preparation of PmFB, ~20 mg was used for fiber type analysis and the remaining sample was frozen for future work. The sequence of microbiopsy and Bergstrom sampling was randomized across participants.

### Preparation of permeabilized muscle fibers (PmFB)

This technique is partially adapted from previous methods (Kuznetsov et al., 1996; Tonkonogi et al., 1998) and has previously been described (Anderson et al., 2007; Perry et al., 2011, 2012). Briefly, small portions (~25 mg) of muscle were dissected from each biopsy and placed in ice-cold BIOPS, containing (in mM): 50 MES, 7.23 K_2_EGTA, 2.77 CaK_2_EGTA, 20 imidazole, 0.5 dithiothreitol (DTT), 20 taurine, 5.77 ATP, 15 PCr, and 6.56 MgCl_2_·6 H_2_O (pH 7.1). The muscle was trimmed of connective tissue and fat and divided into several small muscle bundles (~2–7 mm, 2–5 mg wet weight). Each bundle was gently separated along the longitudinal axis with a pair of anti-magnetic needle-tipped forceps under magnification (Zeiss 2000, Germany). Bundles were then treated with 30 μg/ml saponin in BIOPS and incubated on a rotor for 30 min at 4°C. Saponin at 30 μg/ml has previously been shown to optimize respiration in human skeletal muscle (Kane et al., 2011). Saponin is a mild, cholesterol-specific detergent that selectively permeabilizes the sarcolemmal membranes while keeping mitochondrial membranes, which contain little cholesterol, intact (Veksler et al., 1987; Kuznetsov et al., 2008). PmFB designated for pyruvate supported H_2_O_2_ emission (described below) were also treated with 1 μM CDNB during the permeabilization process to remove endogenous glutathione and better isolate emission kinetics, as previously described (Treberg et al., 2010; Fisher-Wellman et al., 2013). Following permeabilization, the PmFB were washed in either MiR05 containing (in mM): 0.5 EGTA, 10 KH_2_PO_4_, 3 MgCl_2_·6 H_2_O, 60 K-lactobionate, 20 Hepes, 20 Taurine, 110 sucrose and 1 mg/ml fatty acid free BSA (pH 7.1) for respiration experiments, or Buffer Z containing (in mM): 105 K-MES, 30 KCl, 10 KH_2_PO_4_, 5 MgCl_2_·6 H_2_O, 1 mM EGTA, 5 mg/ml BSA, (pH 7.1) for H_2_O_2_ emission experiments at 4°C until measurements were initiated (< 30 min).

### Mitochondrial respiration in permeabilized muscle fiber bundles

High-resolution O_2_ consumption measurements were conducted in 2 ml of respiration medium (MiR05) using the Oroboros Oxygraph-2k (Oroboros Instruments, Corp., Innsbruck, Austria) with stirring at 750 rpm. Respiration medium contained 20 mM Cr to saturate mitochondrial creatine kinase (Saks et al., 1991, 1994, 1995; Walsh et al., 2001; Anmann et al., 2006). For ADP-stimulated respiratory kinetics, either 5 mM glutamate or 5 mM pyruvate, accompanied by 0.5 mM malate, were added as complex I substrates (via generation of NADH to saturate electron entry into complex I) followed by a titration of submaximal ADP (225 and 750 μM) and maximal ADP (5 mM). Succinate (20 mM) was then added under state three conditions to saturate electron entry into complex II. Cytochrome *c* was added to test for mitochondrial membrane integrity, with all experiments demonstrating < 10% increase in respiration. All experiments were conducted in either the presence (+) or absence (−) of 5 μM BLEB in the assay media which allowed for the comparison of respiratory kinetics in contracted or relaxed states for each biopsy technique. Each protocol was completed before the oxygraph chamber [O_2_] reached 150 μM. Polarographic oxygen measurements were acquired in 2 s intervals, with the rate of respiration derived from 40 data points, and expressed as pmol/s/mg wet weight. PmFB were weighed in ~1.5 ml of tared BIOPS (ATP-containing relaxing media) to prevent rigor that occurs when weighing PmFB in open air (unpublished observations).

### Mitochondrial H_2_O_2_ emission in permeabilized muscle fiber bundles

Mitochondrial hydrogen peroxide (H_2_O_2_) emission was determined fluorometrically (QuantaMaster 40, HORIBA Scientific, Edison, New Jersey) in a quartz cuvette with continuous stirring at 37°C, in 1 mL of Buffer Z supplemented with 10 μM Amplex Ultra Red, 0.5 U/ml horseradish peroxidase, 1 mM EGTA, 20 mM creatine, and 40 U/ml Cu/Zn-SOD1. Either 10 mM succinate or 10 mM pyruvate were added to stimulate H_2_O_2_ emission followed by a titration of ADP in step wise increments to progressively attenuate emission. All measurements were made in the presence of 5 μM BLEB to allow for the comparison of H_2_O_2_ emission in the relaxed state between biopsy techniques. Due to tissue limitations with the microbiopsy, no H_2_O_2_ emission measurements were made in -BLEB. The rate of H_2_O_2_ emission was calculated from the slope (F/min), after subtracting the background, from a standard curve established with the same reaction conditions and normalized to fiber bundle wet weight as described above.

### Microscopic imaging of PmFB conformation

Images of Bergstrom and microbiopsy PmFB conformation were captured prior to, and immediately following respiration experiments. Photographs were captured with a Samsung Galaxy S5 camera (South Korea) placed face-down on a transparent acrylic surface at 2.4-4X zoom focused on PmFB placed in a culture dish containing MiRO. Video recordings with the same device were made to document the change in conformation of both Bergstrom PmFB and microbiopsy PmFB +BLEB and -BLEB following the addition of 2 mM ADP (with 5 mM pyruvate and 2 mM malate) in MiR05 buffer. The media temperature was maintained at 33–37°C by a pre-heated metal block.

### Immunofluorescence analysis of fiber type

Immediately after removal from the needles, muscles were embedded in O.C.T. compound (Tissue Tek), frozen in liquid nitrogen-cooled isopentane and stored at −80°C, until analysis. 10 μm thick cryosections were cut with a cryostat (Thermo Electronic), maintained at −20°C and transferred onto static-free microscope slides. Immunofluorescent detection of myosin heavy chain isoforms was performed as previously described (Bloemberg and Quadrilatero, 2012). In addition, fiber membranes were visualized by staining for dystrophin (DSHB, University of Iowa, Iowa City, USA). Fiber type composition analysis was performed on image composites by counting all fibers across the entire cross section. Slides were visualized with an Axio Observer Z1 fluorescent microscope equipped with an AxioCam HRm camera and associated AxioVision software (Carl Zeiss, Germany).

### Statistics

Results are expressed as mean ± SEM. The level of significance was established at *P* < 0.05 for all statistics. The D'Agostino—Pearson omnibus normality test was first performed to determine whether data resembled a Gaussian distribution. Due to failed normality, Friedman's non-parametric One-way ANOVA test was performed for all respiration data. When a significant F-ratio was obtained, Dunn's multiple comparisons *post-hoc* analysis was performed. Paired *t*-tests were performed to assess differences amongst biopsy techniques with respect to H_2_O_2_ emission rates in presence of BLEB as well as fiber type proportions.

## Results

### Comparison of ADP-induced contraction in skeletal muscle PmFB

**Figure 1:**
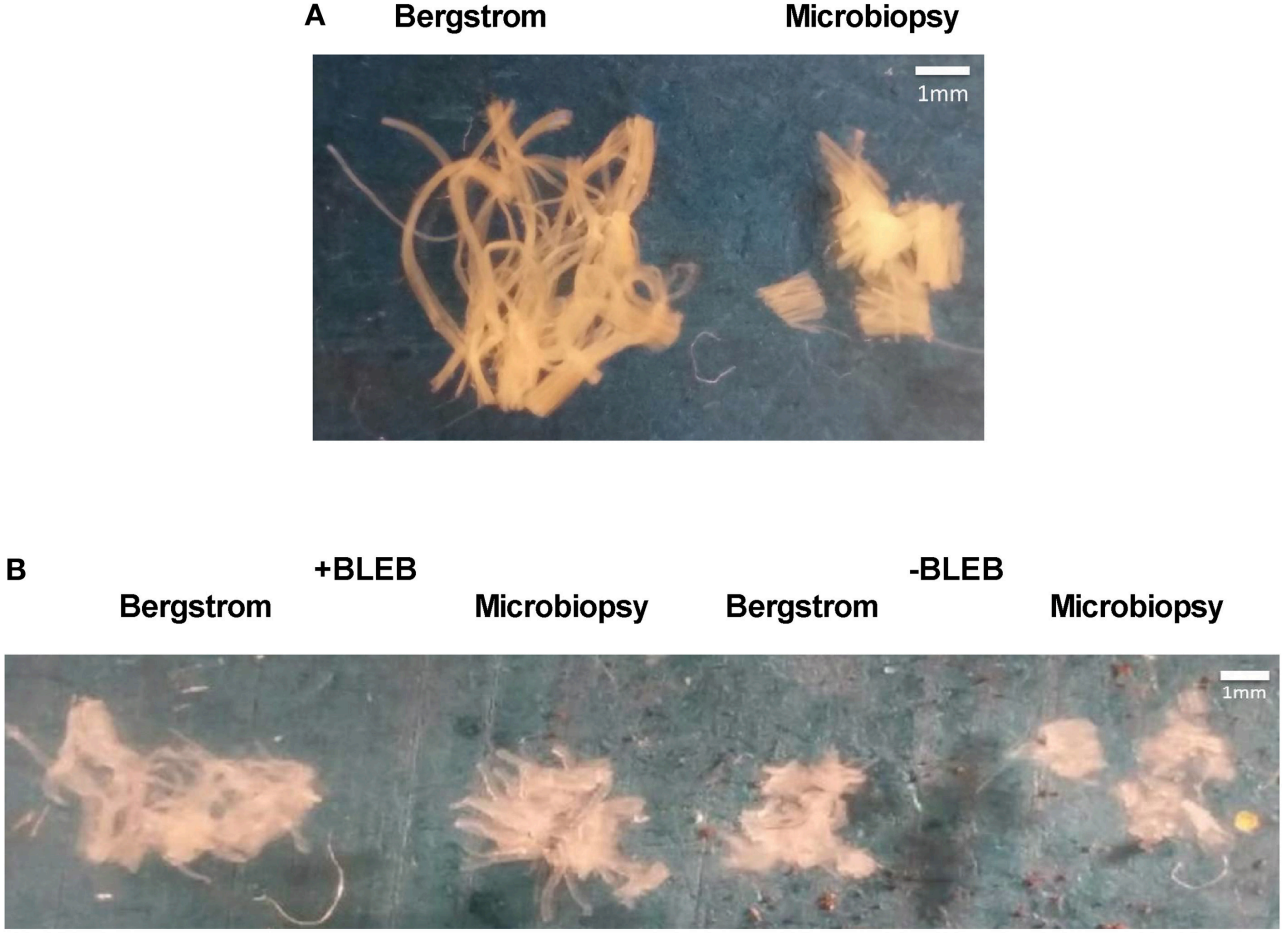
**Effect of apparent ADP-stimulated respiration on human PmFB conformation**. PmFB conformation was imaged **(A)** prior to exposure to ADP-stimulated respiration in Bergstrom and microbiopsy PmFB (4x zoom) and **(B)** following 45–60 min of ADP-stimulated respiration in the presence (+) and absence (−) of BLEB (2.4x zoom).

We first compared qualitative appearances of PmFB conformation from Bergstrom vs. microbiopsy sample in the relaxed state prior to ADP-stimulated respiration (Figure 1A). Bergstrom PmFB appeared to have longer and more connected individual fibers forming a more cohesive bundle while microbiopsy PmFB had much shorter individual fibers. Following titrations of ADP over a period of 45–60 min at 37°C in +BLEB or −BLEB, PmFB were retrieved from the respirometer chambers and imaged. The Bergstrom PmFB in −BLEB appeared less separated and more compact than those from +BLEB, suggesting greater ADP-induced rigor in the absence of BLEB (Figure 1B). However, 41% of microbiopsy PmFB from −BLEB disintegrated into multiple pieces, many of which were so small that they could not be removed from the oxygraph chamber for imaging (Figure 1B) whereas nearly all PmFB in +BLEB for either biopsy could be removed after the experiments.

**Figure 2:**
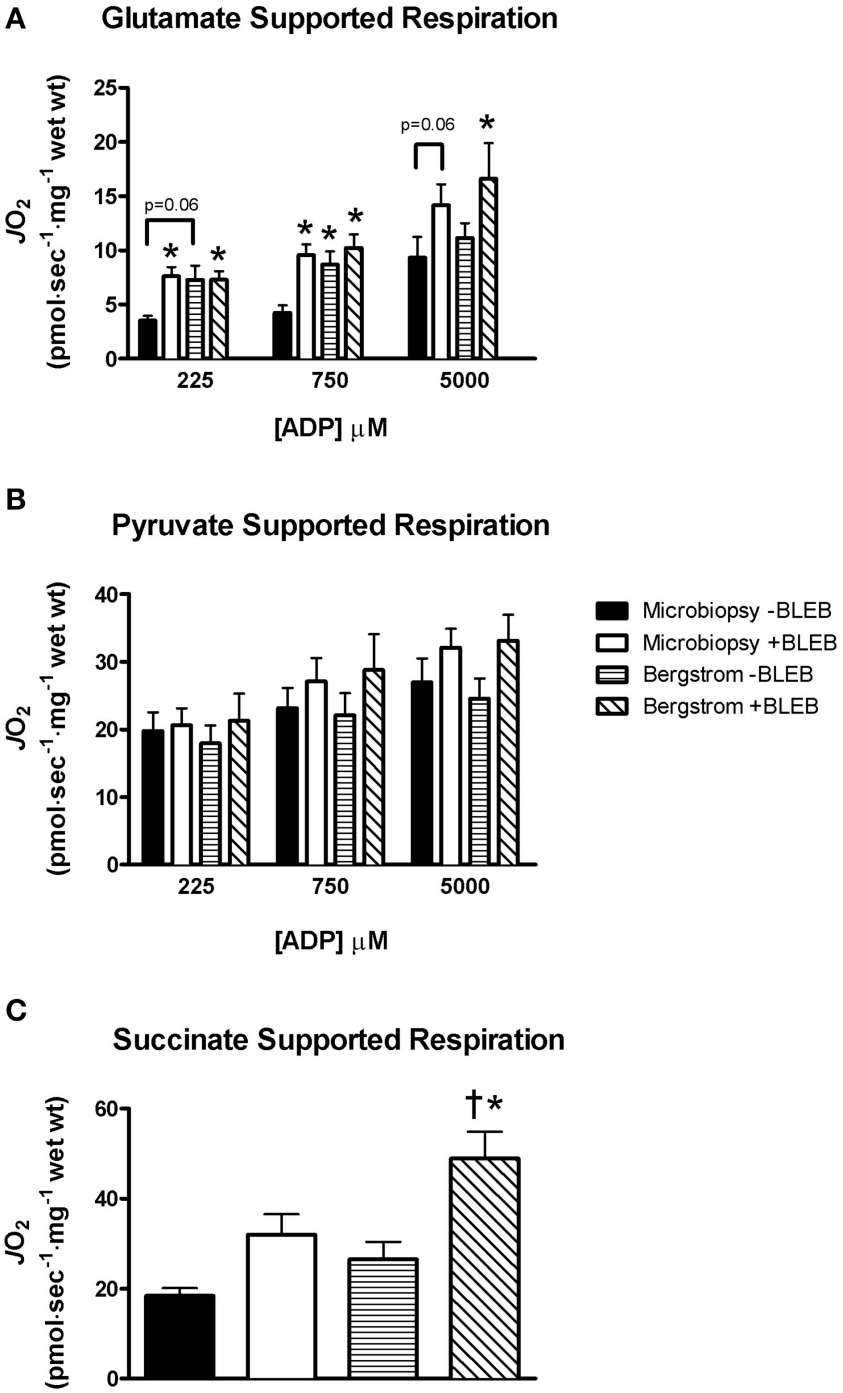
**Effect of apparent ADP-induced contraction on mitochondrial respiration in PmFB**. ADP-stimulated respiration rates were supported by **(A)** glutamate (5 mM), **(B)** pyruvate (5 mM), and **(C)** succinate (20 mM) in microbiopsy and Bergstrom PmFB. Results represent means ± S.E.M.; *n* = 8–11; ^*^P < 0.05 compared to microbiopsy—BLEB within the same [ADP] condition, ^†^*P* < 0.05 compared to Bergstrom—BLEB.

**Figure 3:**
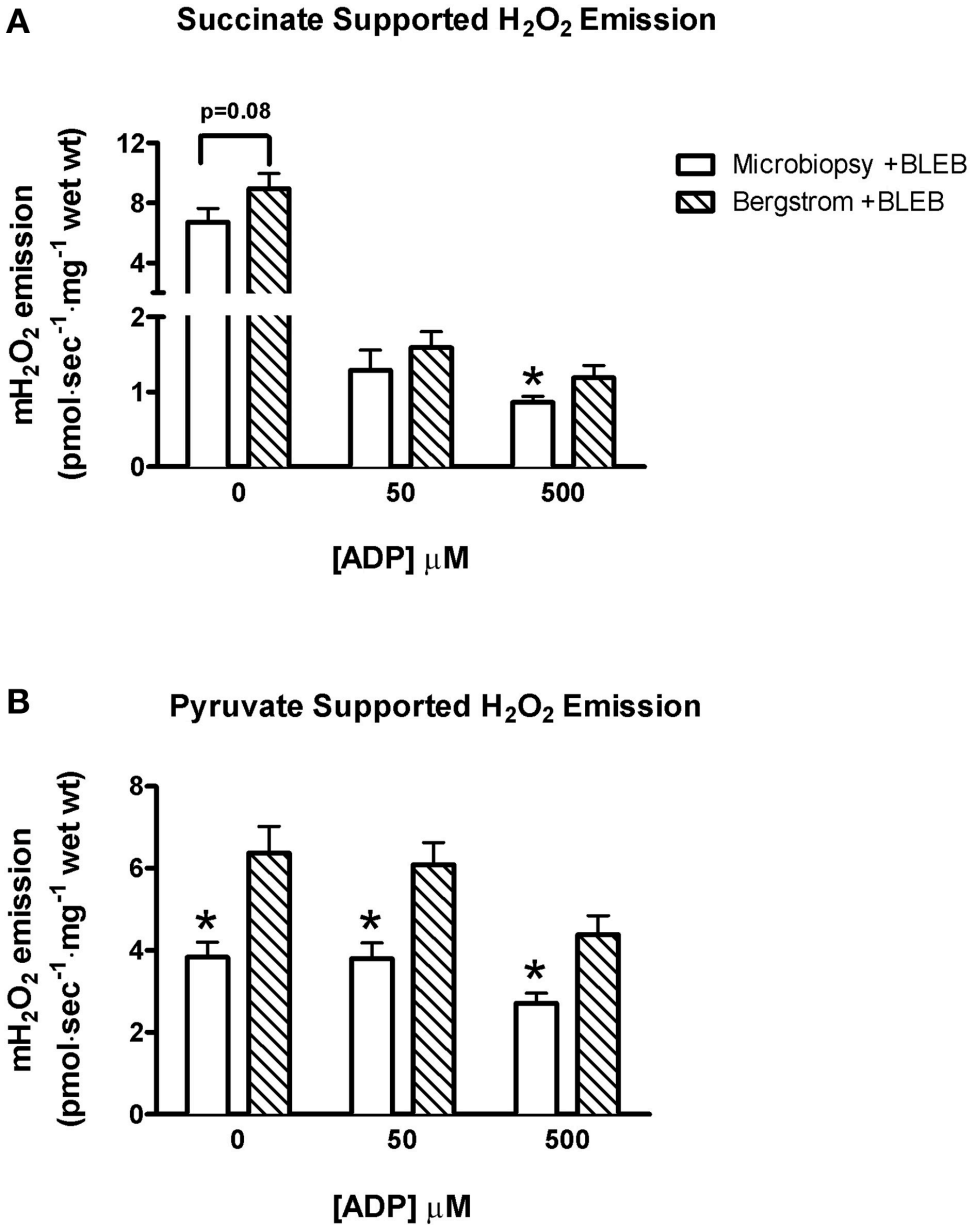
**H_2_O_2_ emission rates of PmFB sampled from microbiopsy and Bergstrom biopsy techniques in the presence of 5 μM BLEB**. Mitochondrial H_2_O_2_ emission rates at varying concentrations of ADP titrated after stimulation by 10 mM **(A)** succinate and **(B)** pyruvate in PmFB from microbiopsy and Bergstrom samples. Results represent means ± S.E.M.; *n* = 11; ^*^P < 0.05 compared to Bergstrom of same [ADP].

**Figure 4:**
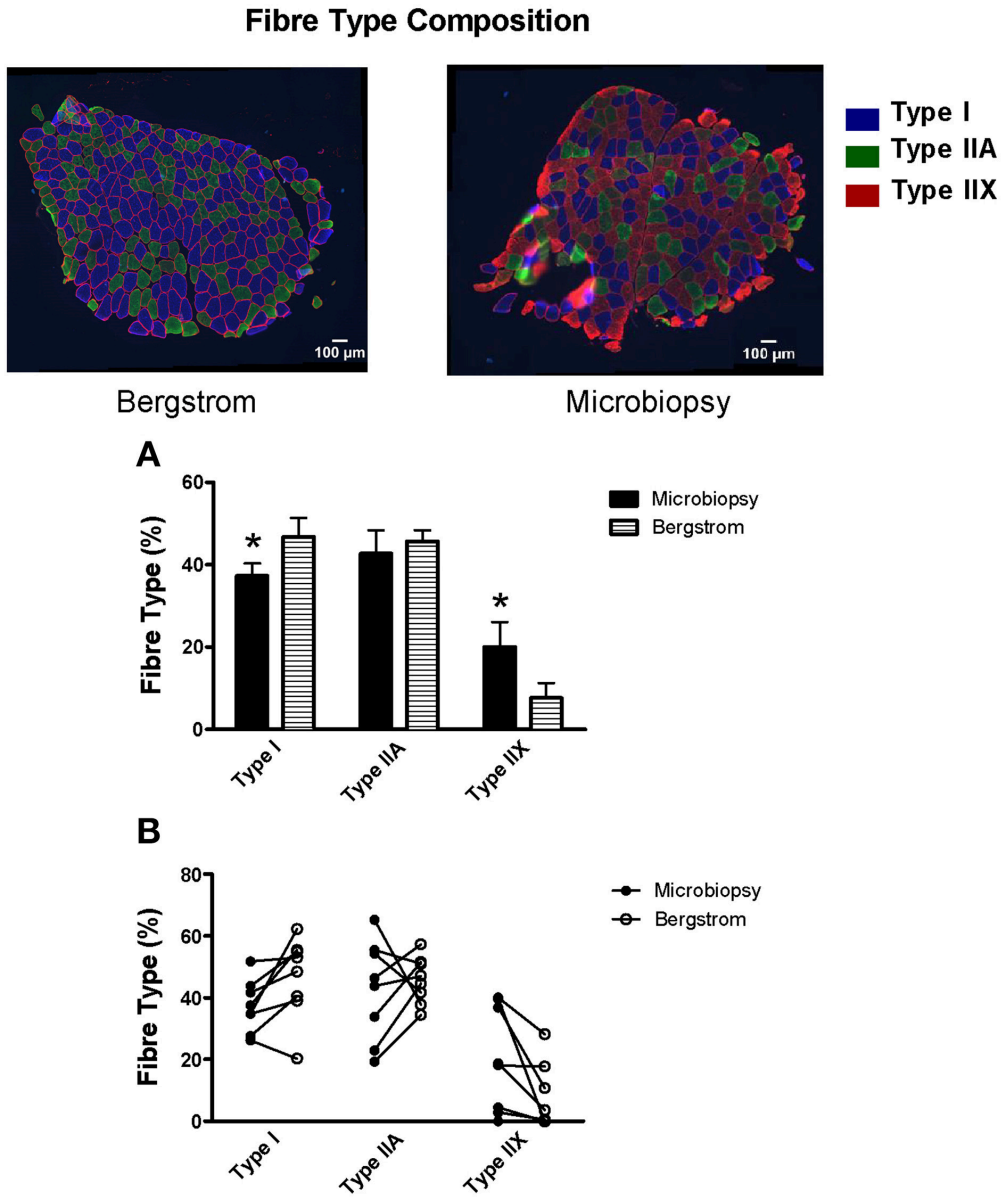
**Fiber type composition of microbiopsy and Bergstrom muscle samples**. Relative percentage of fiber type from samples obtained using the microbiopsy and Bergstrom biopsy technique are represented with blue indicating type I, green indicating type IIA and red indicating type IIX. Results represent **(A)** means ± S.E.M and **(B)** individual data; *n* = 8–9; ^*^P < 0.05 compared to Bergstrom of same fiber type.

Using video capture in separate PmFB, we observed a progressive contraction in response to ADP in −BLEB from Bergstrom (Supplementary Video Figure 1) as previously reported (Perry et al., 2011, 2012) that was also apparent in PmFB from microbiopsy (Supplementary Video Figure 3). ADP-stimulated contraction was not observed +BLEB from either biopsy type (Supplementary Video Figures 2, 4). All videos were 4 min in duration and may therefore underestimate the effect of ADP on contraction that occurs during typical respiratory protocols lasting >30 min.

While force production was not measured, these results suggest the change in PmFB conformation in response to ADP was contraction, given the response was prevented by BLEB.

### BLEB increases ADP-stimulated respiration in a substrate specific manner

In the absence of BLEB, microbiopsy respiration was 51% lower than Bergstrom when supported by glutamate and malate at 225 μM ADP (*p* = 0.06) and 51% lower at 750 μM ADP (*p* < 0.05) with no differences at 5 mM ADP (Figure 2A). When 5 μM BLEB was added to the assay media, the impaired glutamate/malate-supported respiration in microbiopsy PmFB was increased at all ADP concentrations compared to microbiopsy −BLEB (Figure 2A). Furthermore, there were no significant differences in respiration between microbiopsy +BLEB, Bergstrom +BLEB or −BLEB (Figure 2A) indicating BLEB normalized microbiopsy respiration to both Bergstrom conditions. Contrary to the findings with glutamate/malate-supported respiration, there were, surprisingly, no differences in any condition during pyruvate-supported respiration between microbiopsy and Bergstrom (Figure 2B). BLEB increased succinate-supported respiration in Bergstrom by 84–166% vs. both microbiopsy −BLEB and Bergstrom −BLEB, but did not significantly affect microbiopsy respiration (Figure 2C).

### Lower mitochondrial H_2_O_2_ emission rates in microbiopsy PmFB

Due to tissue limitations, mitochondrial H_2_O_2_ emission was measured in both biopsies only in the presence of BLEB. Succinate-induced H_2_O_2_ emission was 37% greater in Bergstrom +BLEB vs. microbiopsy +BLEB at 500 μM ADP (*p* < 0.05) and trended greater at 0 μM ADP (*p* = 0.08), with no difference at 50 μM ADP (Figure 3A). Similarly, pyruvate induced H_2_O_2_ emission rates were 51–66% greater in Bergstrom vs. microbiopsy at all ADP concentrations (*p* < 0.05, Figure 3B).

### Fiber type analysis reveals differences in myosin expression between microbiopsy and bergstrom biopsies

Immunoflourescent analyses of fiber type composition revealed microbiopsy samples have a significantly lower proportion of type I fibers vs. Bergstrom (37.3 vs. 46.7%, *p* < 0.05, Figure 4) and a significantly higher proportion of type IIX fibers (20.1 vs. 7.6%, *p* < 0.05, Figure 4). However, no differences in relative proportion of type IIA fibers were observed (42.7 vs. 45.7%, Figure 4).

## Discussion

This investigation demonstrates that myosin inhibition in PmFB is a novel solution to resolving the reduction in respiratory kinetics observed in microbiopsy samples of human skeletal muscle. Specifically, in −BLEB, PmFB from Bergstrom biopsies appeared more compacted and less separated following ADP-stimulated respiration whereas PmFB from microbiopsies had a greater occurrence of disintegration into fragments. This *in vitro* change in morphology lowered glutamate-supported respiratory kinetics in PmFB from microbiopsies moreso than in Bergstrom. As hypothesized, the lower respiration in microbiopsy PmFB were increased with the myosin II ATPase inhibitor Blebbistatin, suggesting ADP stimulated contraction of PmFB in -BLEB was responsible for the change in morphology in response to ADP and reduction in respiration vs. Bergstrom. Microbiopsies also contained fewer type I and greater type IIX fibers. However, this does not explain the lower glutamate-supported respiration given kinetics were similar with other substrates in all conditions. Mitochondrial H_2_O_2_ emission rates were detectable in samples obtained using the microbiopsy technique but absolute values were lower relative to Bergstrom, even in the presence of BLEB. Collectively, these results highlight the importance of considering needle sampling approaches when assessing mitochondrial bioenergetics in human skeletal muscle and clearly demonstrate that microbiopsies can be used to assess mitochondrial respiration in PmFB when apparent ADP-induced contraction is prevented by BLEB.

### Preventing microbiopsy ADP-induced contraction *in vitro* improves glutamate-supported respiration

Microbiopsy sampling is seen as an attractive alternative to Bergstrom biopsies in part, because of the smaller diameter of the needle. However, concerns about the length of fibers obtained from a smaller diameter needle have been raised (Isner-Horobeti et al., 2014). Previous work demonstrating a positive correlation between needle diameter and maximal rates of respiration in PmFB suggested that this impairment in respiration is related to fiber length (Isner-Horobeti et al., 2014). This notion is supported by differences in bundle morphology observed in the present study (Figure 1A), which demonstrates shorter myofiber length and a disorganized conformation in microbiopsy relative to Bergstrom. Previous work with Bergstrom PmFB revealed that spontaneous ADP-induced contraction results in drastic changes in conformation including rigor and fragmentation (Perry et al., 2011, 2012). This followed previous work showing ADP causes rigor in PmFB from rodent skinned cardiac fibers (Ventura-Clapier and Vassort, 1985). Given the small fibers in microbiopsies pose a challenge to preparing intact PmFB, it is not surprising that this same apparent contraction results in more consistent and complete disintegration of PmFB (Figure 1B). However, similar to previous findings in Bergstrom PmFB (Perry et al., 2011, 2012), BLEB prevents this apparent ADP-induced contraction and partially retains microbiopsy PmFB conformation, myofiber length and structural integrity (Figure 1B). This result demonstrates that BLEB corrects the observed reductions in glutamate-supported respiration reported previously (Isner-Horobeti et al., 2014) and therefore permits the use of microbiopsies for respiratory assessments in PmFB.

Surprisingly, these disintegration-related impairments in respiration were substrate-specific. While we generally observed similar degrees of contraction regardless of the substrate utilized (unpublished findings), this disintegration did not appear to have an impact on pyruvate-supported respiration rates given respiration was similar between both biopsy techniques with or without BLEB present (Figure 2B). However, succinate-supported respiration was increased by BLEB in Bergstrom PmFB which was greater than the rate obtained in microbiopsies PmFB. Speculating on the substrate-specific differences in respiration between biopsies is difficult but might suggest contraction exerts a regulatory influence on specific metabolic pathways through mechanisms that might be retained in PmFBs but are yet to be realized. While the reason for these substrate-specific differences is unclear, this finding highlights the necessity of considering all experimental parameters when choosing a biopsy technique and the subsequent protocols that will be used. Given BLEB improved glutamate-supported respiration and did not impair kinetics with pyruvate, we suggest that BLEB should be supplemented in assay media during respiratory assessments supported by any of these substrates. In our hands, this approach allays concerns regarding the use of microbiopsies of PmFB as reported previously (Isner-Horobeti et al., 2014).

It should be noted that previous work demonstrating impaired respiration in microbiopsy PmFB was performed at 22°C (Isner-Horobeti et al., 2014) whereas the present study was conducted at 37°C. While we cannot conclude whether BLEB would normalize microbiopsy respiration to Bergstrom at 22°C, we have previously shown BLEB is more effective at influencing respiratory kinetics in human PmFBs at 37°C vs. lower temperatures (Perry et al., 2011). Nevertheless, it may be preferable to conduct PmFB respiration experiments at or near body temperature given that temperature is a critical regulator of metabolic control *in vitro* and *in vivo*.

### Mitochondrial H_2_O_2_ emission is lower in microbiopsy vs. bergstrom PmFB

It is tempting to speculate that the lower H_2_O_2_ emission in microbiopsies were a result of fewer mitochondria related to the greater proportion of type IIX fibers and reduced type I fibers. However, succinate- and pyruvate/malate-supported H_2_O_2_ emission has been shown to be higher in rat red gastrocnemius muscle [predominantly type IIA and IIX fibers (Bloemberg and Quadrilatero, 2012)] than soleus [predominantly type I fibers (Bloemberg and Quadrilatero, 2012)] when normalized to muscle mass (Anderson and Neufer, 2006). Surprisingly, this apparent inverse relationship between H_2_O_2_ emission and assumed mitochondrial content is opposite to our observations of lower H_2_O_2_ emission in microbiopsies with greater type IIX content. Thus, based on previous findings (Anderson and Neufer, 2006), it would appear that factors independent of mitochondrial content and fiber type might explain the lower H_2_O_2_ emission in microbiopsies.

Anecdotally, it is known that PmFB must be separated as much as possible without losing bundle integrity in order to optimize the detection of H_2_O_2_ emission. Specifically, we found Bergstrom fibers can be separated to a greater degree than microbiopsy PmFB without losing integrity (Figure 1A). This potentially allows for greater diffusion of substrates and ADP within the fiber, and may indicate that H_2_O_2_ emission measurements are more sensitive to the degree of separation than perhaps previously recognized. Hence, it is possible that the relatively lower degree of separation required to maintain integrity in microbiopsy PmFB may result in under-estimations of H_2_O_2_ emission.

These measurements were made in the presence of BLEB and suggest that preventing PmFB contraction does not normalize microbiopsy PmFB H_2_O_2_ emission to Bergstrom. Whether BLEB still improved this measurement in comparison to untreated PmFB is uncertain as we were not able to compare ±BLEB due to tissue limitations in microbiopsies. Ultimately, H_2_O_2_ emission is detected quite clearly in microbiopsy +BLEB which does not rule out the use of microbiopsies for this measurement. Nevertheless, potential underestimations relative to Bergstrom should be considered.

### Fiber type differences in microbiopsies vs. Bergstrom: does this influence respiratory assessments?

An intriguing difference in fiber type composition existed between samples obtained from the two needles. Specifically, 7/8 subjects demonstrated an increase in percentage of type I fibers and a decrease in percentage of type IIX fibers in the Bergstrom sample relative to microbiopsy. It is well-established that the human vastus lateralis has a heterogeneous composition (Johnson et al., 1973; Komi and Karlsson, 1978; Lexell et al., 1984; Taylor and Bachman, 1999; Staron et al., 2000). In fact, the distribution varies mainly as a function of depth in that the superficial vastus lateralis contains more type II fibers whereas a greater proportion of type I fibers are found in deeper portions of the muscle (Lexell et al., 1983; Lexell and Taylor, 1989). Given the microbiopsy technique was performed at a shallower depth in the present study (~30° insertion angle relative to the skin), these differences in fiber type composition might be attributed to the difference in needle depth. However, we cannot rule out potential contributions of the longitudinal location between the two needles given the shallow insertion angle of the microbiopsy generally sampled at a location more proximal to the knee.

It is tempting to speculate that the lower glutamate-supported respiration with microbiopsies was due to the lower type I and higher type IIX content, as discussed above for H_2_O_2_ emission. However, there were no differences in pyruvate or succinate-supported respiration between either needle in ±BLEB. This suggests the fiber type differences in human muscle were too small to influence the kinetics even though large differences in fiber type in rodents have been shown to influence respiration (Kuznetsov et al., 1996). Nonetheless, it would appear that the small differences in fiber type do not explain the lower glutamate-supported respiratory kinetics or H_2_O_2_ emission in microbiopsy PmFB -BLEB. We cannot rule out that such composition differences between needles could influence other metabolic assessments.

Ultimately, the differences in fiber type between sampling methods does not necessarily pose a problem for skeletal muscle metabolic assessments *per se*. Rather, the interpretation of results should be made in the context of sample fiber type composition inherent in each sampling method. Of course, fiber type composition could differ within a sampling approach due simply to variations in sampling depth or longitudinal consistency within trials, highlighting the need for consistency in sampling procedure regardless of needle type.

### Selecting a muscle biopsy approach: important considerations

Categorizing one procedure as superior to the other must be considered in the context of specific parameters. The obvious advantage of the Bergstrom method is a larger sample yield with typical ranges of ~50–250+ mg depending, in part, on the diameter of the needle, the use of suction and the number of cuts. However, we obtained a typical yield of ~70–90 mg (and up to 120 mg) with microbiopsy by rotating the needle ~30 degrees to uncut areas of muscle between five separate cuts while applying light pressure on the skin prior to each cut. A fast procedure is required to minimize blood contamination of muscle (< 60 s including removal of muscle after each cut). Additionally, participant comfort level must also be considered. While microbiopsies are smaller in diameter, these five separate cuts also require separate insertions into the same cannula guide and therefore take longer to obtain. Conversely, the Bergstrom procedure permits multiple cuts over several seconds with a single insertion. However, 8 out of 11 participants reported preferring the microbiopsy technique over the Bergstrom when questioned following both procedures. Furthermore, reduced risks of infection are sometimes marketed by commercial suppliers of microbiopsy needles due specifically to the fact that they are disposable, thereby eliminating the need for subsequent sterilization. However, proper sterilization of Bergstrom needles obviously ameliorates this concern. These claims must also be balanced with the fact that the single-stage cut with microbiopsies requires removal of skeletal muscle from the needle with sterile forceps prior to re-insertion of the needle into the muscle for additional cuts. This requires considerable care in preventing contamination of the needle in between samples from the same entry site.

It should also be noted that our conclusions are specific to a 14 gauge needle from a specific supplier. Other models of disposable microbiopsy needles could influence the results based on needle sharpness or length of time required to obtain a sample given some needles do not have a cannula “guide” that accelerates the procedure as in the present study. Likewise, we have not compared the influence of multiple gauges, although previous work suggests a positive correlation exists between PmFB respiration and needle diameter (Isner-Horobeti et al., 2014). Finally, experimental procedures requiring intact muscle fibers should carefully consider the requirement for specific fiber lengths independent of sample size. Overall, the advantages and disadvantages to both procedures must be considered within the context of specific study designs rather than focusing on needle diameter and sample yield as the sole criteria for selecting a needle.

## Conclusions

In conclusion, this investigation reveals that PmFB prepared from 14 gauge microbiopsies provide similar glutamate-supported respiratory kinetics to Bergstrom samples when assay media are supplemented with 5 μM BLEB to prevent apparent ADP-induced contraction and maintain PmFB integrity. The similar pyruvate-supported respiration between biopsy approaches further suggests that microbiopsies can be used to measure respiratory kinetics in PmFB, although Bergstrom +BLEB provided the greatest rate of succinate-supported respiration. However, observed reductions in H_2_O_2_ emission and altered type I:type IIX fiber types should be considered when adopting microbiopsies. These findings highlight critical experimental parameters to consider when selecting biopsy needles and underscores the importance of preventing ADP-induced PmFB contraction by myosin inhibition with Blebbistatin *in vitro*.

## Author contributions

MH, BG, JQ, DK, and CP. contributed to study design. MH, SR, PT, AN, HT, RL, and CP conducted clinical trials and/or experiments while MH, BB, JQ, DK, and CP analyzed and interpreted the data. MH and CP were the primary writers of the manuscript while all authors contributed to critical interpretation and manuscript preparation. All authors approved the final manuscript.

## Funding

Funding was provided to CP by National Science and Engineering Research Council (#436138-2013) with infrastructure supported by Canada Foundation for Innovation, the Ontario Research Fund and the James H. Cummings Foundation. DK was supported by NSHRF REDI (#2012-8799). MH was supported by a Canadian Institutes of Health Research CGS-M scholarship.

### Conflict of interest statement

The authors declare that the research was conducted in the absence of any commercial or financial relationships that could be construed as a potential conflict of interest.
