# Supplementary material for: Mitochondrial Bioenergetics and Fiber Type Assessments in Microbiopsy vs. Bergstrom Percutaneous Sampling of Human Skeletal Muscle
Source: Front Physiol. 2015 Dec 18;6:360. doi: 10.3389/fphys.2015.00360 (PMC4683189; doi:10.3389/fphys.2015.00360)
Supplement: Supplementary file 5 [file DataSheet1.DOCX]

Supplementary Material

**Mitochondrial bioenergetics and fibre type assessments in microbiopsy vs Bergstrom percutaneous sampling of human skeletal muscle**

M.C. Hughes^*^, S.V. Ramos, P.C. Turnbull, A. Nejatbakhsh, B.L. Baechler, H. Tahmasebi, R. Laham, B.J. Gurd, J. Quadrilatero, D.A. Kane, and C.G.R. Perry^†^

^†^**Correspondence:** Corresponding Author: C.G.R Perry, cperry@yorku.ca

# Supplementary Figures and Tables

**Supplementary Figure 1.** ADP-induced contraction of human vastus lateralis PmFB obtained using the Bergstrom biopsy technique in MiRO at 33°C - 37°C in the presence of pyruvate and malate (5 mM/0.5 mM) and 5 µM BLEB. 1 second represents ~ 15 seconds of recorded video. Video began ~5 seconds following initial exposure to 37°C.

**Supplementary Figure 2.** The effect of 5 µM BLEB on ADP-induced contraction of human vastus lateralis PmFB obtained using the Bergstrom biopsy technique in MiRO at 33°C - 37°C in the presence of pyruvate and malate (5 mM/0.5 mM). 1 second represents ~ 15 seconds of recorded video. Video began ~5 seconds following initial exposure to 37°C.

**Supplementary Figure 3.** ADP-induced contraction of human vastus lateralis PmFB obtained using the microbiopsy technique in MiRO at 33°C - 37°C in the presence of pyruvate and malate (5 mM/0.5 mM). 1 second represents ~ 15 seconds of recorded video. Video began ~5 seconds following initial exposure to 37°C.

**Supplementary Figure 4.** The effect of 5 µM BLEB on ADP-induced contraction of human vastus lateralis PmFB obtained using the microbiopsy technique in MiRO at 33°C - 37°C in the presence of pyruvate and malate (5 mM/0.5 mM). 1 second represents ~ 15 seconds of recorded video. Video began ~5 seconds following initial exposure to 37°C.
